# Spatial predictions of tree density and tree height across Mexico forests using ensemble learning and forest inventory data

**DOI:** 10.1002/ece3.10090

**Published:** 2023-05-21

**Authors:** Aylin Barreras, José Armando Alanís de la Rosa, Rafael Mayorga, Rubi Cuenca, César Moreno‐G, Carlos Godínez, Carina Delgado, Maria de los Ángeles Soriano‐Luna, Stephanie George, Metzli Ileana Aldrete‐Leal, Sandra Medina, Johny Romero, Sergio Villela, Andrew Lister, Rachel Sheridan, Rafael Flores, Thomas W. Crowther, Mario Guevara

**Affiliations:** ^1^ Department of Forest and Rangeland Stewardship Colorado State University Fort Collins Colorado USA; ^2^ Centro de Geociencias Universidad Nacional Autónoma de México Juriquilla Mexico; ^3^ Comisión Nacional Forestal (CONAFOR) Zapopan Mexico; ^4^ US Forest Service, International Programs Washington District of Columbia USA; ^5^ Institute of Integrative Biology ETH Zurich Zürich Switzerland; ^6^ Department of Environmental Sciences University of California Riverside California USA; ^7^ U.S. Salinity Laboratory, Agricultural Research Service United States Department of Agriculture Riverside California USA

**Keywords:** ensemble machine learning, forest inventory, spatial prediction, tree density, tree height

## Abstract

The National Forestry Commission of Mexico continuously monitors forest structure within the country's continental territory by the implementation of the National Forest and Soils Inventory (INFyS). Due to the challenges involved in collecting data exclusively from field surveys, there are spatial information gaps for important forest attributes. This can produce bias or increase uncertainty when generating estimates required to support forest management decisions. Our objective is to predict the spatial distribution of tree height and tree density in all Mexican forests. We performed wall‐to‐wall spatial predictions of both attributes in 1‐km grids, using ensemble machine learning across each forest type in Mexico. Predictor variables include remote sensing imagery and other geospatial data (e.g., mean precipitation, surface temperature, canopy cover). Training data is from the 2009 to 2014 cycle (*n* > 26,000 sampling plots). Spatial cross validation suggested that the model had a better performance when predicting tree height *r* ^2^ = .35 [.12, .51] (mean [min, max]) than for tree density *r* ^2^ = .23 [.05, .42]. The best predictive performance when mapping tree height was for broadleaf and coniferous‐broadleaf forests (model explained ~50% of variance). The best predictive performance when mapping tree density was for tropical forest (model explained ~40% of variance). Although most forests had relatively low uncertainty for tree height predictions, e.g., values <60%, arid and semiarid ecosystems had high uncertainty, e.g., values >80%. Uncertainty values for tree density predictions were >80% in most forests. The applied open science approach we present is easily replicable and scalable, thus it is helpful to assist in the decision‐making and future of the National Forest and Soils Inventory. This work highlights the need for analytical tools that help us exploit the full potential of the Mexican forest inventory datasets.

## Graphical abstract

Due to the challenges involved in collecting data exclusively from field surveys, there are spatial information gaps in the Mexican forest inventory. We performed wall‐to‐wall spatial predictions of tree height and tree density in 1‐km grids, using ensemble machine learning across each forest type. The applied open science approach we present is easily replicable and scalable, thus it is helpful to assist in the decision‐making and future of the National Forest and Soils Inventory.

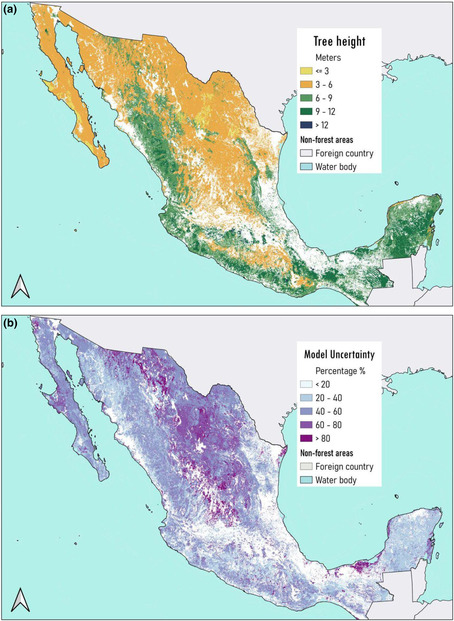

## 1 INTRODUCTION

Forest inventories continuously monitor the status of forested ecosystems through the implementation of field campaigns for data collection and subsequent analysis (Smith, 2002). As forests play a key role in maintaining ecologic stability, national forest inventories (NFI) are playing an increasingly‐important role in driving academic and governmental decision making (Saarela et al., 2020). Traditionally, forest inventories have been used for the development of environmental policy, such as land and timber management strategies at regional and national scales. NFI later began to contribute to international reports. For example, Mexico's National Forest and Soils Inventory (INFyS) is a pillar for the measurement, reporting, and verification system (MRV): it is the foundation for the national inventory of greenhouse gasses (GHG) emissions in the Land Use, Land‐Use Change, and Forestry (LULUCF) sector and for the national forest reference emissions level (FREL). MRV and FREL are components of a carbon accounting system used by the United Nations to incentivize practices that lower carbon emissions (Mitchell et al., 2017). NFI usually focus on collecting field data over large geographic areas. Developing analytical tools that enhance the accessibility and understanding of nation‐wide forest inventory data is critical for democratizing information about forest structure at national and international scales.

The National Forestry Commission of Mexico (CONAFOR) has been in charge of implementing the INFyS from 2004 to the present. The INFyS is a national program in which a stratified, systematic sample of permanent ground plots is used to measure trees (e.g., height, diameter at breast height, count) and site (e.g., forest type, site class, topographic data) variables across all forest lands every 5 years (CONAFOR, 2017). The INFyS data have been collected across a range of climatic zones within Mexico including tropical forests, coniferous and broadleaf forests, cloud mountain forests, mangroves, and arid and semi‐arid regions. Thereby, Mexico's NFI data are extremely valuable and useful at national and international scales, and in academia. INFyS data are openly available at https://snmf.cnf.gob.mx/datos‐del‐inventario/. In recent years, the INFyS has been working towards the development and update of data analysis methodologies such as geospatial analysis for the mapping of forest structure and also in overcoming the technical challenges that come with sample‐based forest inventories.

Forest inventories based on a statistical sample are used to estimate mean or total amounts of forest inventory attributes within the population of interest (Tomppo, Haakana, et al., 2008). However, field surveys can be costly, time consuming and logistically‐challenging. Collecting data exclusively from field surveys can result in designs that do not satisfy the statistical assumptions and can lead to limited sample sizes due to the phenomenon of non‐response, which occurs when field plots that were part of the design cannot be accessed. Improper management of nonresponse can produce bias or increase uncertainty when generating estimates (McRoberts et al., 2005). Emerging satellite and machine learning (ML) technologies give us the opportunity to build standardized analytical models, based on NFI field‐data, which can help with problems associated with non‐response (e.g., fill data gaps) and produce maps that serve for multiple purposes (Tomppo, Olsson, et al., 2008).

Mapping forest attributes through the integration of NFI and remote sensing data has been widely applied to better visualize national‐scale estimates, reduce uncertainty, and improve dataset robustness (Haakana et al., 2019; Ohmann et al., 2014; Saarela et al., 2020; Tomppo et al., 2010). This approach has played a key role in modeling national estimates of forest structure such as aboveground biomass (AGB) as well as attributes such as forest age (Saarela et al., 2020; Schumacher et al., 2020). To obtain accurate spatial predictions of forest attributes, many studies employ ML models using a multivariate approach (Khaledian & Miller, 2020; Li et al., 2020; Soriano‐Luna et al., 2018; Wadoux et al., 2020). ML is a field of artificial intelligence (AI), and one of its main objectives is to identify and model relationships between dependent data (such as forest inventory attributes) and independent data (such as remote sensing), and apply these models to generate predictions in a semi‐autonomous approach (James et al., 2013a). The performance of different types of ML models often varies when modeling forest attributes. For example, spatially explicit estimates of AGB varied by as much as 19% when performing linear (LM), generalized additive (GAM) and random forest (RF) empirical models in a temperate forest in central Mexico (Soriano‐Luna et al., 2018). The three fitted AGB models performed well when predicting AGB spatial distribution, but GAM was better for representing AGB variations across the landscape. Thus, different ML models yield different results and studies use multiple models or algorithms to identify the best solutions for predicting forest attributes or specific response variables, as no silver bullets exist in ecological modeling (Qiao et al., 2015).

One commonly used set of ML approaches used to perform spatial prediction are ensemble learners, which integrate multiple ML models and algorithms (Holloway & Mengersen, 2018). Ensemble ML models are used in mapping forest attributes because they offer improvements in accuracy to independent algorithms (Healey et al., 2018). Examples of popular ensemble ML algorithms include RF (Breiman, 2001), which applies a bagging method to create a forest of uncorrelated decision trees, another one is Super Learner, which applies a stacked method and uses cross‐validation to estimate the performance of multiple ML models (Polley & van der Laan, 2010). The latter has been shown to outperform the individual algorithms used to build the model (Davies & van der Laan, 2016; Taghizadeh‐Mehrjardi et al., 2021).

The main goal of this study is to develop a methodological framework in which CONAFOR can generate country‐level maps of INFyS forest attributes. Specifically, this involves operationalizing methods based on integrating field data with remote sensing data in an ensemble ML framework to map forest attributes. We are starting with tree height and tree density, as these are key components of forest structure and can be useful to provide information that helps with the impacts of nonresponse, and in the estimation of AGB, carbon storage and forest productivity over time (Humagain et al., 2017; Pirotti, 2010; Selkowitz et al., 2012). Accurate spatial predictions of such structural variables are fundamental for the management and conservation of forest ecosystems, as they are important constituents in the study of land‐atmosphere interactions, carbon cycling, assessment of fire hazards and timber volume estimation (Chopping et al., 2008; Selkowitz et al., 2012). By developing workflows and products based on INFyS data, this study aims to support CONAFOR in generating information that will be used by decision makers to manage forests more effectively, preserve the country's forest patrimony, and improve national and international reporting associated with MRV and FREL. We envision this methodology could be further applied for several other forest attributes such as AGB, carbon storage and timber volume, among others, and improve Mexico's national estimates of other relevant forest attributes.

## 2 MATERIALS AND METHODS

### 2.1 Study area

**FIGURE 1:**
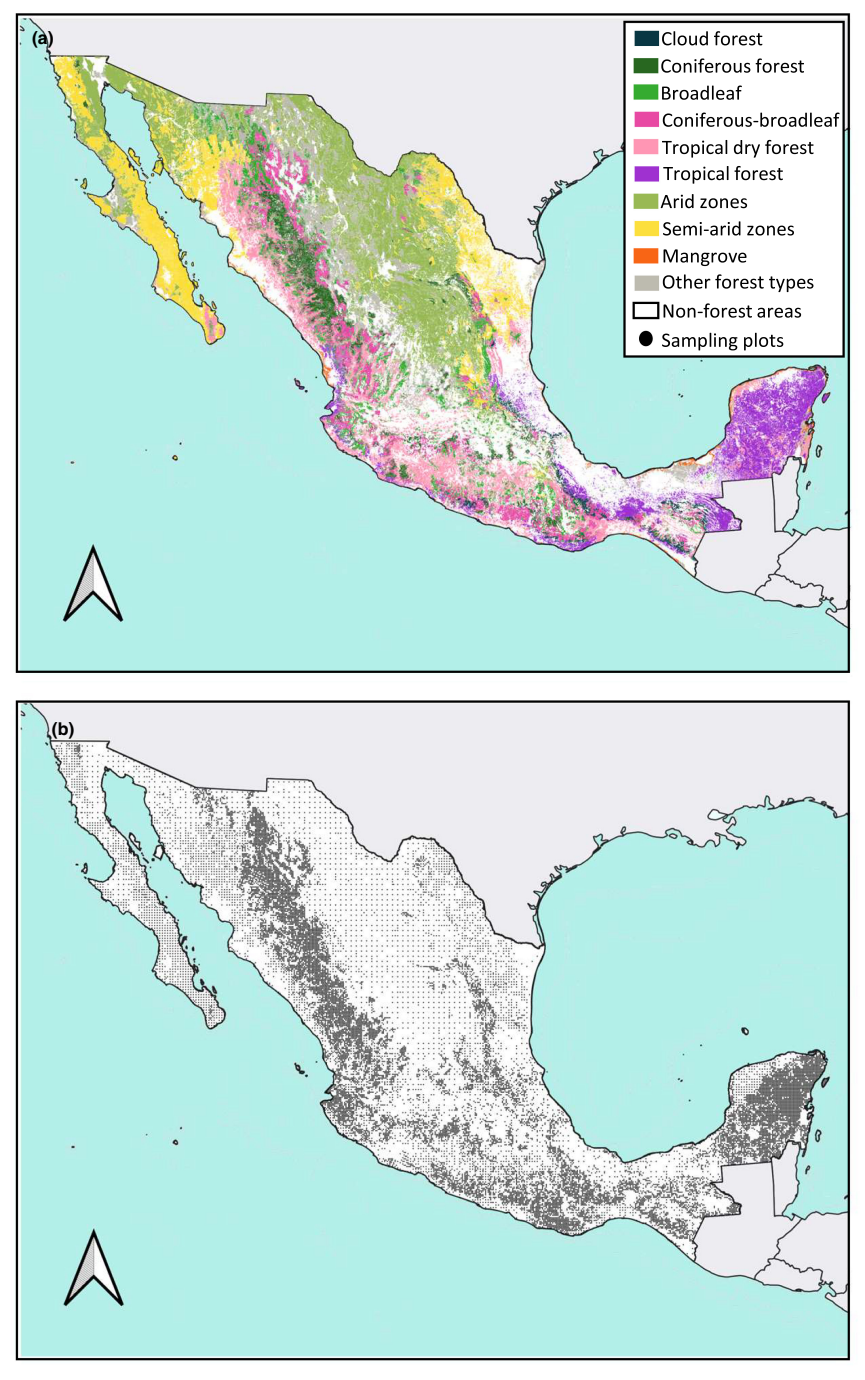
(a) Map of Mexico forest types and (b) map of INFyS sampling plots (black dots). Prepared from the Land Use and Vegetation map, scale 1:250,000, Series VI, Instituto Nacional de Geoestadística y Geografía (CONAFOR, 2017; INEGI, 2017).

The study was conducted at a national scale and included all forest types in Mexico (Figure 1a). The country is located between latitudes 32° and 14°N, where the Nearctic and Neotropical biogeographic zones converge. Due to its geographical location, the territory has complex topographic and climatic characteristics (CONABIO, 1998). From the arid zones in the northwest to the humid rainforest in the southeast, forest ecosystems in Mexico are very diverse. They comprise a vast variety of vegetation, having tree heights ranging between 60 m in coniferous forests to 1.3 m in xerophilous scrubs (CONAFOR, 2017). Tree species of economic interest include mahogany (*Swietenia macrophylla*) and cedar (*Cedrela odorata*), which are typical of tropical forests.

### 2.2 Mexico National Forest and Soils Inventory data

Tree height and tree density models were developed using plot level data collected between 2009 and 2014 and obtained from the INFyS database. The sampling design considered a total of 26,220 plots distributed across the Mexican territory during 2009–2014 (Figure 1b), however 9.5% of the total plots were categorized as inaccessible sites (CONAFOR, 2017). The number of sampled plots for each forest ecosystem were 2606 for coniferous forests, 4111 for coniferous‐broadleaf, 3249 for broadleaf forest, 483 for cloud mountain forest, 3724 for tropical forest, 1466 for tropical dry forests, 240 for arid zones, 1334 for semiarid zones, and 157 for mangrove forests. Data are available from the Environmental Data Initiative (EDI): https://doi.org/10.6073/pasta/4620375aea631ab6a09cb573c7bf8aff (Barreras et al., 2022) and at the official web page https://snmf.cnf.gob.mx/datos‐del‐inventario/.

Sampled plots are distributed across all land cover types, ecological stages, and land tenure classes (e.g., private, social, government). Plot distribution is accomplished through systematic, pre‐stratified sampling with 5 × 5 km spacing in temperate and tropical forests (which included natural and cultivated forests), 10 × 10 km in dry and semi‐arid vegetation communities and 20 × 20 km in arid vegetation strata (includes samples of succulents). These strata are derived from a forest type map created by the Mexican government (Figure 1a; INEGI, 2017). The plot is considered a cluster design with four circular primary subplots, three of which are configured in a triangular array around a central subplot (Figure S2). Primary subplots, where trees with a diameter at breast height (dbh, 1.3 m above ground) ≥7.5 cm are measured, have a radius of 12.56 m and are 400 m^2^ in area; spacing between adjacent primary subplot centers is ~45 m (CONAFOR, 2017). Tree height was measured at each primary subplot for all trees with a dbh ≥7.5 cm; then, an average tree height value was estimated for each plot. The height of trees was measured from the base of the tree to the tip of the canopy, including dead branches. In general, the predominant height categories in more than 90% of the data (all ecosystems) are between 5 and 10 m, which indicates that they are semi‐regular masses (CONAFOR, 2017). For the purpose of this study, a tree is defined as those greater than or equal to 7.5 cm dbh.

### 2.3 Remotely sensed data as model predictors

As a cloud‐based platform, Google Earth Engine (GEE) provides easy access to an extensive catalog of satellite imagery and other geospatial data for scientific, business, and government users (Gorelick et al., 2017). We obtained a combination of topographic, climatic, and vegetation derived variables with pixel sizes of 1000 m for the period of 2009–2014 from GEE. In this manner, we assembled a nation‐wide geospatial dataset of 39 covariates for tree height and tree density predictions.

The number of potential predictor layers was reduced to 6 through a dimension reduction process, guided by a random forest (RF) prediction for each target variable (tree height and density) and the set of 39 predictors. We used the randomForest package v4.7‐1.1 for R v4.1.0, a method that implements feature bagging to implement a prediction (Breiman, 2001). We then calculated predictors' importance using the importance function from the RF package. Variables with the higher percentage increase in mean squared error (%IncMSE) were selected as most important (Table S1). After identifying the most important predictors for each target variable, we used the ClustOfVar package v1.1 for R v4.1.0 to group strongly correlated predictors that potentially bring the same information and can cause overfitting in the data (Chavent et al., 2017). We selected the final set of predictors based on the results of both dimension reduction techniques. All covariates were resampled to 1000 m. The resampling was done with conventional bilinear interpolation as implemented in GEE. Data are available from Zenodo under the name “Nationwide geospatial dataset of environmental covariates at 1km resolution in Mexico” (https://doi.org/10.5281/zenodo.7130164; Barreras & Guevara, 2022).

### 2.4 Spatial prediction using LANDMAP

To perform the spatial predictions, we used an ensemble ML approach called Super Learner, a meta‐model that linearly combines the predictions from multiple models (e.g., kernel‐based, tree‐based, linear based or neural network based) (Polley & van der Laan, 2010; van der Laan et al., 2007). We applied the Super Learner algorithm as implemented in the LANDMAP package v0.0.14 for R v4.1.0, which provides a strategy for automated mapping (Hengl et al., 2018, 2021; Polley & van der Laan, 2010; RStudio Team, 2021) (https://github.com/Envirometrix/landmap). Methods implemented in the model ensemble were decision trees‐based methods (random forest), kernel‐based methods (support vector machines), methods based on neural networks (NNET), and generalized linear models (GLM). We assumed that different methods describe relationships in our data in a different manner. The model was run on each forest type separately.

We took advantage of the geographical distances in our training data and used the oblique geographic coordinates technique to incorporate spatial dependence as an additional predictor, as used by previous studies (Møller et al., 2020). To eliminate covariances and dimensionality, predictors were converted to principal components before running the Super Learner model. Therefore, we transform the relationship between covariates in the multivariate space to vectors of numbers that are not related to each other, that is, they are orthogonal in the statistical space. We expressed the uncertainty of our estimates in percentage form as the range of the 68% prediction intervals divided by their mean for each pixel, as performed by Viscarra Rossel et al. (2014). We used a fivefold spatial cross validation (spCV) approach to assess the predictive accuracy of our modeling framework. spCV differs from standard cross‐validation by accounting for the spatial autocorrelation between data points used for model training and validation; this way, training points are statistically independent from validation points (Brenning, 2012; James et al., 2013b). Ignoring spatial autocorrelation in data can lead to an overoptimistic evaluation of predictive power (Ploton et al., 2020). The spCV yields model independent residuals required to compute map quality indicators such as: the coefficient of determination (*r* ^2^) and root mean square error (RMSE). To further compare model accuracy, we use conditional quantile plots (Carslaw, 2015).

## 3 RESULTS

### 3.1 Descriptive statistics of sampled inventory data

Maximum field measurements of tree height were 36 m and found in coniferous forests and coniferous‐broadleaf forests. Mean tree heights measured in the field ranged from 5 to 10 m, with the exception of arid and semi‐arid zones, where trees had an average height of ~4 m (Figure S4).

Mean field estimates of tree density were higher in tropical forests, with an average of ~790 trees per ha. Generally, the other forest types had an average of ~400 to 500 trees per ha, even mangroves which had a relatively small number of sampled plots (157). Arid and semi‐arid zones had an average of ~88 and ~162 trees per ha, respectively.

### 3.2 Model predictors

**FIGURE 2:**
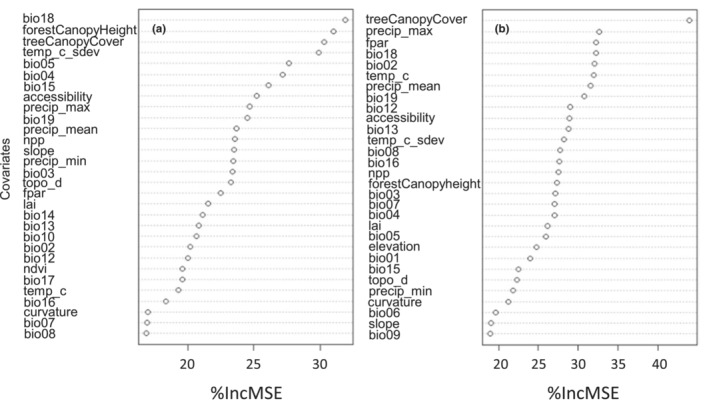
Covariate importance for selected target variables (a) tree height and (b) tree density based on the percentage increase in mean squared error (%IncMSE).

The parameter used for measuring covariate importance was the percentage increase in mean squared error (%IncMSE), as shown in Figure 2. The covariate “bio18”, which accounts for the precipitation of warmest quarter, is the most important covariate for tree height, while treeCanopyCover is the most important when predicting tree density (see Figure S1).

Results from the second dimension‐reduction technique, which consisted in an unsupervised cluster analysis, showed the predictors related to each other (Figure S1). Predictors such as forestCanopyCover and fpar (fraction of photosynthetic active radiation) were among the first 3 most important for tree density (Figure 2b); however, they turned out to be variables that share a high correlation according to the cluster analysis. We decided to use only one of them as a predictor for our models. We followed this understanding to choose six predictors with high importance as well as with low correlation between each other to further avoid statistical redundancy in our predictions.

The final set of predictors chosen for both target variables were (1) tree canopy cover (treeCanopyCover) (Hansen Global Forest Change v1.8 2000–2020) (Hansen et al., 2013), (2) mean precipitation (precip_mean) (CHIRPS Pentad: Climate Hazards Group InfraRed Precipitation with Station Data v2.0) (Funk et al., 2015), (3) SRTM‐derived topographic diversity (topo_d) (Theobald et al., 2015), (4) Mean land surface temperature standard deviation (temp_c_sdev) (AG100: ASTER Global Emissivity Dataset 100‐meter V003) (Hulley et al., 2009, 2012, 2015; Hulley & Hook, 2008, 2009, 2011; NASA JPL, 2014), (5) temperature seasonality (bio04), and (6) precipitation of warmest quarter (bio18), both from WorldClim V1 (Hijmans et al., 2005; Table S2).

### 3.3 Model summaries and evaluation

Descriptive statistics for each forest‐type predicted tree height and density maps are presented in Table 1. The mean values of predictions are generally aligned with those of the plots. Tree height models with the highest *r* ^2^ values were coniferous‐broadleaf forest, broadleaf forest and mangroves (Table 1).

**TABLE 1:** Summary statistics of tree height and tree density predictions for all forest ecosystems in Mexico.

Forest type	Tree height			Tree density		
	Mean (m)	*r* ^2^	RMSE	Mean (trees/ha)	*r* ^2^	RMSE
Coniferous forest	8.22	.4	3.35	457.42	.24	311.01
Coniferous and broadleaf forests	7.51	.50	2.78	425.79	.21	294.95
Broadleaf forest	5.8	.51	2.25	372.58	.21	300.72
Cloud forest	9.47	.12	2.69	460.42	.17	356.64
Tropical forest	8.93	.26	1.88	741.57	.42	469.56
Tropical dry forest	6.15	.40	1.78	501.58	.30	377.07
Arid zones	3.78	.16	1.16	65.79	.05	101.70
Semi‐arid zones	4.11	.35	1.30	149.85	.23	181.54
Mangroves	6.40	.45	2.94	545.46	.16	426.36

Abbreviations: *r* ^2^, coefficient of determination; RMSE, root mean square error.

Moreover, the model performed tree density predictions with higher *r* ^2^ in tropical forests and tropical dry forests. Despite tropical forests having the highest *r* ^2^, it also was the ecosystem with the highest RMSE.

On average, predicted tree height ranged between 4 and 9 m in all forest ecosystems (averaged from all pixel values). Cloud forest, arid and semi‐arid zones had smaller *r* ^2^ for both target variables, which could be related to the smaller amount of sampled data in these ecosystems. However, arid and semi‐arid zones seemed to have the smallest errors in both tree height and tree density predictions.

**FIGURE 3:**
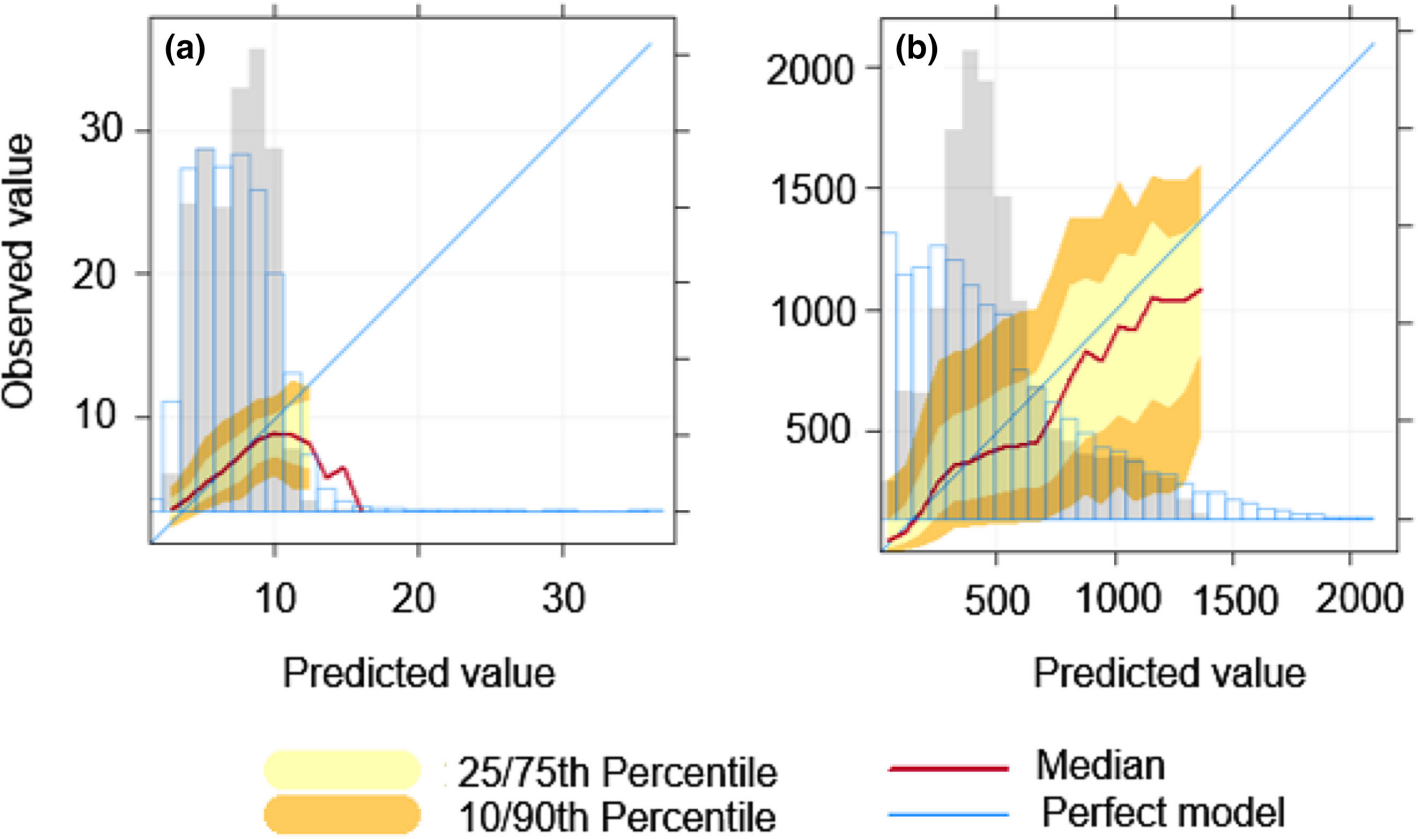
Conditional quantile plots for the observed versus predicted values of (a) tree height and (b) tree density. For each plot the blue line shows the results for a perfect model. The red line shows the median values of the predictions and corresponding observations. The yellow shading shows the predicted quantile intervals, for example the 25/75th and the 10/90th. A perfect model would lie on the blue line and have a very narrow spread. The histogram shows the counts of predicted values (gray bars) and observed values (blue outlined bars) (Carslaw, 2015).

According to the conditional quantile plots, the model had a better predictive performance for tree height than tree density (Figure 3). Conditional quantiles plots examine how well predictions agree with observations. The data are divided into equally spaced bins. For each predicted value, the corresponding value of the observations is identified and the median, 25/75th and 10/90 percentile (quantile) are calculated for that bin (Carslaw, 2015). As shown by the median (red line) in Figure 3a, model predictions agree precisely with observation from the range of 1 to ~10 m of tree height. From 10 m onwards, model performance weakens. We also observe that the model slightly overestimated tree height values between 5 and 10 m, as stated by the gray bar on Figure 3a.

Tree density predictions were less precise when compared against observed values. The model had the best performance when predicting tree densities of 500 trees/ha and below (Figure 3b). From 500 trees/ha onwards predictions were less precise, yet they followed a similar pattern as the “perfect model” (blue line). The model overestimated tree density values between 300 and 700 trees/ha; however, predicted values follow a similar distribution to the observed values in both tree height and tree density scenarios. Conditional quantile plots for each forest type can be found in the Figure S5 for tree height predictions and Figure S6 for tree density.

### 3.4 Mapping the tree height and tree density of Mexico forests

**FIGURE 4:**
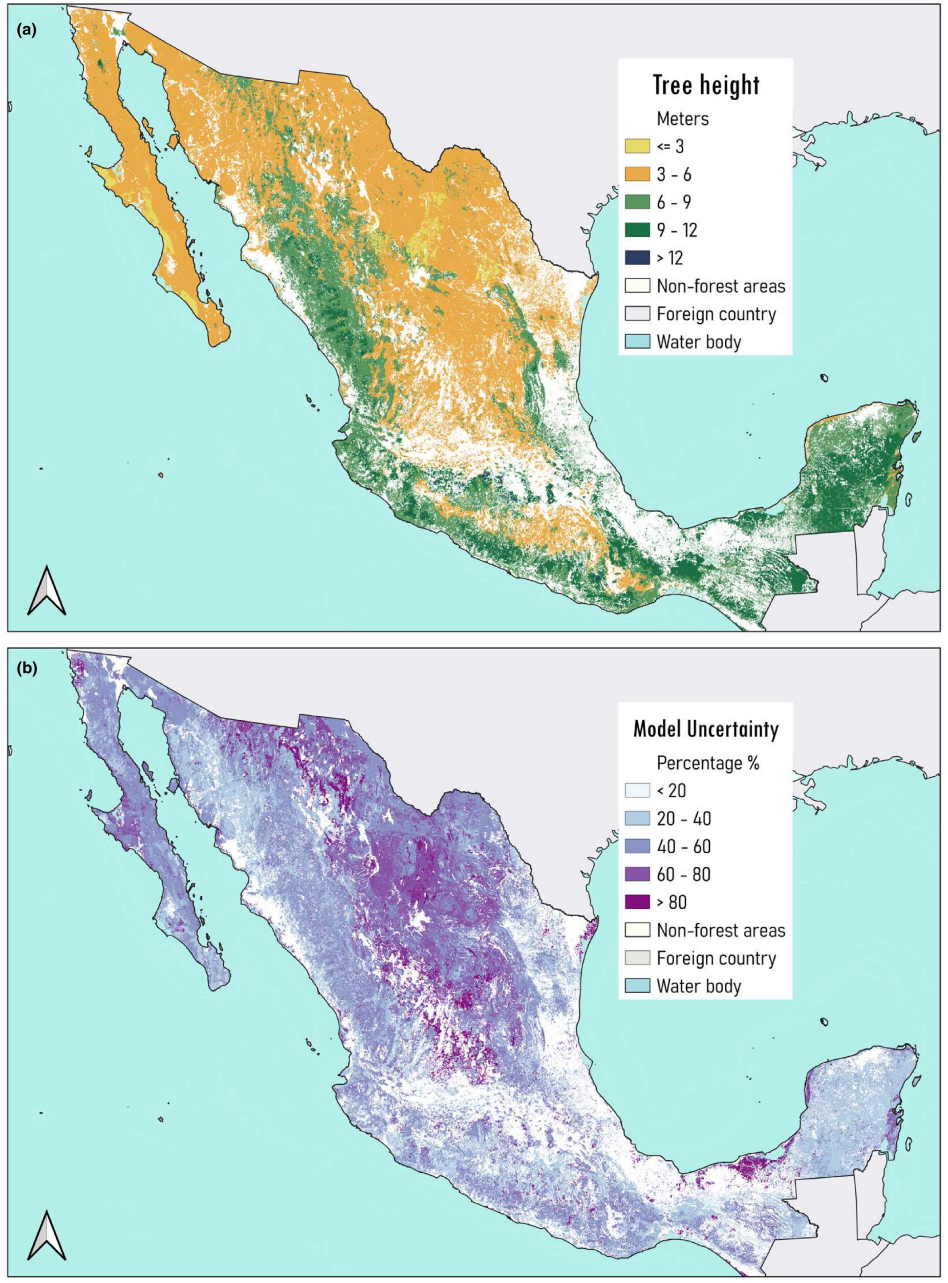
National maps of (a) predicted mean tree height and (b) its associated uncertainty across all Mexico's forest ecosystems.

**FIGURE 5:**
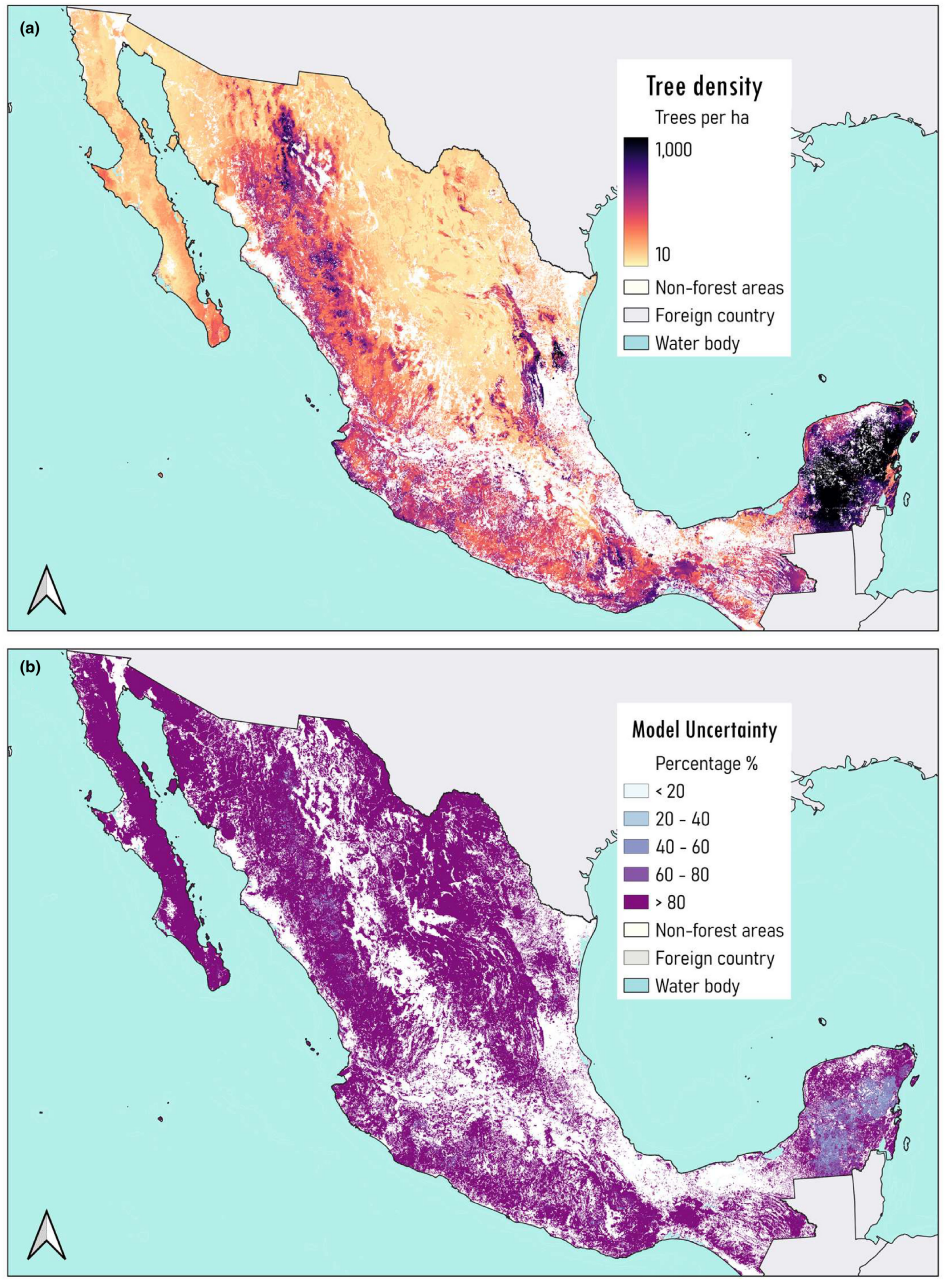
National maps of (a) predicted mean tree density and (b) its associated uncertainty across all Mexico's forest ecosystems.

The models were used to generate spatially continuous national maps of mean tree height (Figure 4) and the total number of trees (Figure 5), both at a 1000‐m resolution, along with their associated uncertainties. At the forest type level, maximum predicted pixel values of tree height were observed in coniferous, coniferous‐broadleaf forests and cloud mountain forests (~20, 14.3 and 12.3 m, respectively). These types of forest ecosystems constitute Mexico's mountain chains Sierra Madre Oriental and Sierra Madre Occidental. Moreover, the smallest tree heights were predicted in arid and semi‐arid zones, having a mean of ~4 m. The model had the highest uncertainty when predicting tree height in arid zones (60%–80%), the latter could be related to the limited sample size we had for that specific forest type (Figure 4b). Lower uncertainty was observed for tropical forest and tropical dry forest.

Tropical forests had the maximum predicted pixel values of tree density (~1457 trees/ha), followed by coniferous‐broadleaf forest (1140 trees/ha), tropical dry forest (1091 trees/ha) and coniferous forest (1079 trees/ha) (Figure 5a). Uncertainty in predictions of tree density was higher compared to tree height (Figure 5b). High uncertainty (e.g., <80%) was observed across all forest types except tropical forest (40%–60%).

## 4 DISCUSSION

Over the last 20 years, CONAFOR has invested significant time and resources to produce forest inventory data that accurately represents all forest ecosystems in Mexico. To further expand the utility of this data, we developed an analytical framework to model, predict, and map forest structural attributes across the country. By exploiting the available remotely sensed data (e.g., mean surface temperature, tree canopy cover, mean precipitation, and topographic diversity) (Gorelick et al., 2017), the ensemble ML method in the LANDMAP package v0.0.14 for R v4.1.0 (Hengl et al., 2021; RStudio Team, 2021), and the openly available INFyS data (CONAFOR, 2017), we have modeled and performed predictions of tree height and tree density across Mexico. Results suggest that the ensemble ML algorithm had a better performance when predicting tree height over tree density (Table 1). In addition to providing numerical estimates, these maps are user‐friendly devices that help users visualize forest structures across Mexico.

Mapping forest attributes along with associated uncertainties at a national scale requires substantial computational resources. Using high resolution covariates (e.g., 30 m) has helped achieve an increase in model predictive ability (Hengl et al., 2021). Here, we had limited computational resources. We decided to simplify our approach by modeling at a 1000‐m resolution and reducing the number of model predictors, thus reducing computing costs and still displaying valuable nation‐wide maps for political and ecological matters. However, it is important to acquire sufficient computational resources for the project's next stage and perform more accurate predictions with high‐resolution covariates.

Remotely sensed predictors such as tree canopy cover, mean precipitation, mean temperature standard deviation, and topographic diversity had the highest importance in predicting both target variables, while maintaining a relatively low correlation between each other. Tree canopy cover ranked the most important predictor for tree density and was the third most important for tree height. Moreover, precipitation of the warmest quarter ranked the most important predictor for tree height. Previous studies have shown that using vegetation traits as model predictors can reduce prediction uncertainty when mapping forest attributes (Saarela et al., 2020). Moreover, Heilman et al., 2022 found a strong positive effect of water‐year precipitation when forecasting tree growth, which is directly related to tree height and density. Results from our feature selection approach agree with the a‐priori understanding of forest structure, its environmental drivers and our conception of ecological modeling.

The range of mean predicted values for tree height were consistent with forest inventory data (~5 to 10 m), suggesting that the super learner model reflected the input data adequately. On average, cloud mountain forest is the ecosystem with the tallest trees in Mexico (Table 1). This particular forest belongs to humid and temperate areas; it has the largest aerial biomass density and the greatest timber volume of all Mexico forest types, but it accounts for only ~1% of the national forest area (Villaseñor & Gual, 2014). According to CONAFOR (2017), more than half of its vegetation is in early stages of succession, with high densities of young trees due to the wide timber exploitation. Nonetheless, cloud forests were among the ecosystems with the less precise predictions (Table 1). Conditional quantile plots indicated that the model had the best predictive performance for broadleaf and coniferous‐broadleaf forests (Figure S5), coinciding with other quality indicators (*r* ^2^, RMSE) results. The model explains ~50% of the variance for both forest types (Table 1).

Globally, ~42% of the planet's trees exist in tropical and subtropical regions (Crowther et al., 2015). Generally, optimal conditions for tree growth are warm temperatures and moisture availability (Leathwick & Austin, 2001). In accordance with this assumption, tropical forests have the highest tree density of all Mexico forest types (maximum values of ~1457 trees/ha). The model best explained tree density variance for tropical forests (~40%) (Table 1). Conditional quantile plots showed the best predictive performance for tropical forests as well (Figure S6), especially in the range of 500–1500 trees/ha. Overall, the model does a good job estimating mean values of tree densities. The highest number of trees can be observed in the Calakmul rainforest area located within the Yucatán Península, in the southeast of Mexico (Figure 5a). The Calakmul rainforest is part of an important ecological gradient, the Mesoamerican Biological Corridor. The conservation of this ecologically important region has been a challenge due to continuous forest disturbances. Tree density can be an indicator of forest degradation on tropical ecosystems (Román‐Dañobeytia et al., 2014), therefore we encourage the long‐term monitoring of tropical forest structure and the improvement of estimation techniques.

Tree height uncertainty map (Figure 4b) shows areas where the model performs poorly, especially in northern areas which consist of arid and semi‐arid ecosystems (>80% uncertainty). These ecosystems have fewer vegetation patches, which leaves less training data for modeling over a considerably large area of Mexico. Moreover, results from tree density predictions here show high RMSE, which is often above 50% of the mean, resulting in a very high uncertainty (<80%) across all forests as observed in Figure 5b. Uncertainty estimates are a tool to understand the sensitivity of the model to variations in the data. They help us identify certain areas that require more data points and finer covariates resolution due to poor modeling accuracy (e.g., areas with high uncertainty) or even suggest trying a different modeling approach. Another limitation we might be encountering with our ML modeling approach is the effects of anthropogenic influence on forest structure, e.g., combining planted with natural forests, which may reduce model accuracy.

We compared our modeling strategy to using geographic coordinates alone as model predictors as suggested by recent studies (Møller et al., 2020). Results showed a reduction of the explained variance for both tree height and tree density models using this approach. Explained variance for tree height decreased from an average of 0.35% to 0.30%, and from an average of 0.23% to 0.15% for tree density. Therefore, even if our strategy had some limitations, it has a better predictive performance compared to other approaches. To improve map quality indicators (e.g., *r* ^2^, RMSE) and uncertainty, we contemplate repeating the exercise by applying the following features: (1) upgrade computational resources to use covariates at a finer resolution (e.g., 30 m), (2) increase data points for the target variables at specific forest types with poor quality indicators (e.g., cloud forests and arid zones) and (3) assessing different spatial prediction models.

Estimating forest structure is critical for projecting Mexican forests growth trajectories under different management scenarios. Continuous improvement in the study design we present here is encouraged in order to enhance the accuracy of predictions. Results of this study can facilitate the understanding of Mexican forest ecosystems by further applying this methodological framework for the mapping of other forest attributes such as AGB, soil and vegetation carbon storage and their associated functional traits. To achieve this, it is important to continue with active forest inventory campaigns that facilitate the estimation of forest structure patterns through time. Data from this study was managed under the FAIR principles for scientific data management by setting up an open‐access online data repository available at the Environmental Data Initiative (EDI): https://doi.org/10.6073/pasta/4620375aea631ab6a09cb573c7bf8aff.

## 5 CONCLUSIONS

Here, we develop a methodological framework for the spatial prediction of forest attributes, which assists the understanding of forest structure and expands institutional and technical capabilities for data analysis within the National Forestry Commission of Mexico. Out of 10 forest ecosystems, our analyses show that the best predictive performance when mapping tree height was in broadleaf and coniferous‐broadleaf forests (model explained ~50% of variance). The best predictive performance when mapping tree density was in tropical forest (model explained ~40% of variance). For tree height, uncertainties in our predictions were below 60% in most forests. Nonetheless, uncertainties were above 80% in most ecosystems for tree density.

Our results suggest that an ensemble learning framework can be used for the spatial prediction of forest attributes and can likely be improved by having a larger number of field observations and model predictors with a finer spatial resolution that reflect the environment of each forest ecosystem. In order to ensure best practices for forest management in Mexico, it is important that governmental and academic institutions work together to develop methodological approaches. This strategy helps improve the quality and transparency of forestry datasets.

## AUTHOR CONTRIBUTIONS

**Aylin Barreras:** Conceptualization (equal); formal analysis (equal); investigation (equal); methodology (equal); resources (equal); validation (equal); visualization (equal); writing – original draft (lead); writing – review and editing (lead). **José Armando Alanís de la Rosa:** Data curation (equal); formal analysis (equal); funding acquisition (equal); methodology (equal); resources (equal); supervision (equal); validation (equal); writing – original draft (supporting). **Rafael Mayorga:** Data curation (equal); formal analysis (equal); investigation (supporting); methodology (supporting); resources (equal); supervision (equal); validation (supporting). **Rubi Cuenca:** Conceptualization (equal); data curation (equal); formal analysis (equal); investigation (equal); methodology (equal); resources (equal); supervision (equal); validation (equal); writing – review and editing (supporting). **César Moreno‐G:** Conceptualization (equal); data curation (equal); formal analysis (equal); resources (equal); supervision (equal); validation (equal); writing – review and editing (supporting). **Carlos Godínez:** Conceptualization (equal); data curation (equal); formal analysis (equal); investigation (equal); resources (equal); supervision (equal); validation (equal); writing – review and editing (supporting). **Carina Delgado:** Conceptualization (equal); data curation (equal); formal analysis (equal); investigation (equal); resources (equal); supervision (equal); validation (equal); writing – review and editing (supporting). **Maria de los Ángeles Soriano‐Luna:** Conceptualization (equal); data curation (equal); formal analysis (equal); investigation (equal); methodology (equal); resources (equal); supervision (equal); validation (equal); writing – review and editing (supporting). **Stephanie George:** Conceptualization (equal); data curation (equal); formal analysis (equal); investigation (equal); resources (equal); supervision (equal); validation (equal); writing – review and editing (supporting). **Metzli Ileana Aldrete‐Leal:** Conceptualization (equal); data curation (equal); formal analysis (equal); investigation (equal); resources (equal); supervision (equal); validation (equal); writing – review and editing (equal). **Sandra Medina:** Conceptualization (equal); data curation (equal); formal analysis (equal); resources (equal); validation (equal); writing – review and editing (supporting). **Johny Romero:** Conceptualization (equal); data curation (equal); formal analysis (equal); resources (equal); validation (equal). **Sergio Villela:** Conceptualization (equal); data curation (equal); formal analysis (supporting); resources (equal); validation (supporting). **Andrew Lister:** Conceptualization (equal); formal analysis (equal); investigation (equal); methodology (equal); resources (equal); supervision (equal); validation (equal); writing – review and editing (equal). **Rachel Sheridan:** Funding acquisition (equal); project administration (equal); resources (equal); supervision (equal); validation (equal). **Rafael Flores:** Conceptualization (equal); funding acquisition (equal); project administration (equal); resources (equal); supervision (equal); validation (equal). **Thomas W. Crowther:** Conceptualization (equal); methodology (equal); supervision (equal); validation (equal); writing – review and editing (supporting). **Mario Guevara:** Conceptualization (lead); data curation (equal); formal analysis (equal); investigation (equal); methodology (lead); supervision (equal); validation (equal); writing – original draft (equal); writing – review and editing (supporting).

## FUNDING INFORMATION

Mario Guevara acknowledges funding support from UNESCO‐IGCP‐IUGS, 2022 (#765), UNAM‐PAPIIT, 2021 (#IA204522) and USDA‐NIFA‐AFRI, USA, 2019 (#2019‐67022‐29696). The authors acknowledge funding support from the US Forest Service International Programs branch, the US Forest Service Northern Research Station Forest Inventory and Analysis Program, the U.S. Agency for International Development (USAID), and the Universidad Nacional Autónoma de México (UNAM).

## CONFLICT OF INTEREST STATEMENT

The authors declare that there is no conflict of interest.

## Supporting information


Appendix S1

## Data Availability

Training forest data: Data available from the Environmental Data Initiative (EDI): https://doi.org/10.6073/pasta/4620375aea631ab6a09cb573c7bf8aff (Barreras et al., 2022). Environmental prediction factors: Nationwide geospatial dataset of environmental covariates at 1 km resolution in “Mexico” (https://doi.org/10.5281/zenodo.7130164; Barreras & Guevara, 2022).
