# Supplementary material for: Spatial predictions of tree density and tree height across Mexico forests using ensemble learning and forest inventory data
Source: Ecol Evol. 2023 May 21;13(5):e10090. doi: 10.1002/ece3.10090 (PMC10200803; doi:10.1002/ece3.10090)

**Supporting information**

Table S1. Predictors importance measure produced by randomForest for tree height and tree density. %IncMSE: percentage increase in mean squared error.

| Predictor | | Tree height | Tree density |
| --- | --- | --- | --- |
| Name | Description | %IncMSE | %IncMSE |
| accessibility | Accessibility to cities | 25.2 | 29.7 |
| aspect | Terrain aspect | 3.5 | 9.1 |
| bio01 | Annual mean temperature | 8.5 | 21.3 |
| bio02 | Mean diurnal range (mean of monthly (max temp - min temp)) | 20.2 | 31.2 |
| bio03 | Isothermality (bio02/bio07) | 23.4 | 25.3 |
| bio04 | Temperature seasonality (Standard deviation * 100) | 27.2 | 27.8 |
| bio05 | Max temperature of warmest month | 27.6 | 28.1 |
| bio06 | Min temperature of coldest month | 13.5 | 19.8 |
| bio07 | Temperature annual range (bio05-bio06) | 16.9 | 25.4 |
| bio08 | Mean temperature of wettest quarter | 16.9 | 21.5 |
| bio09 | Mean temperature of driest quarter | 16.5 | 18.9 |
| bio10 | Mean temperature of warmest quarter | 20.6 | 19 |
| bio11 | Mean temperature of coldest quarter | 12 | 15.8 |
| bio12 | Annual precipitation | 20 | 31.1 |
| bio13 | Precipitation of wettest month | 20.8 | 25.1 |
| bio14 | Precipitation of driest month | 21.2 | 15.7 |
| bio15 | Precipitation seasonality | 26.1 | 26 |
| bio16 | Precipitation of wettest quarter | 18.4 | 27 |
| bio17 | Precipitation of driest quarter | 19.6 | 16.2 |
| bio18 | Precipitation of warmest quarter | 31.9 | 32.3 |
| bio19 | Precipitation of coldest quarter | 24.5 | 33.5 |
| chili | SRTM-derived CHILI (Continuous Heat-Insolation Load Index) ranging from 0 (very cool) to 255 (very warm). | 14.6 | 17.4 |
| curvature | Terrain curvature | 17 | 20.5 |
| elevation | Elevation | 14.1 | 26 |
| forestCanopyHeight | Tree Height | 31 | 27.9 |
| fpar | Fraction of Photosynthetic Active Radiation absorbed by the green elements of a vegetation canopy | 22.5 | 32.3 |
| hillshade | Terrain hillshade | 14.2 | 15.6 |
| lai | One-sided green leaf area per unit ground area in broadleaf canopies; one-half the total needle surface area per unit ground area in coniferous canopies | 21.6 | 28.8 |
| ndvi | Normalized Difference Vegetation Index | 19.6 | 16.5 |
| npp | Net Primary Productivity | 23.6 | 22.9 |
| precip_max | Maximum precipitation | 24.7 | 33.7 |
| precip_mean | Mean precipitation | 23.7 | 35.7 |
| precip_min | Minimum precipitation | 23.4 | 25.3 |
| slope | Terrain slope | 23.5 | 19.7 |
| surfacewater | The frequency with which water was present. | 9.9 | 5 |
| temp_c | Temperature mean | 19.3 | 31.6 |
| temp_c_sdev | Temperature standard deviation | 29.9 | 29.1 |
| topo_d | SRTM-derived topographic diversity | 23.3 | 25.4 |
| TreeCanopyCover | Tree canopy cover for year 2000, defined as canopy closure for all vegetation taller than 5m in height. | 30.3 | 43.8 |

Fig S1. Hierarchical clustering of the covariables using the R function hclustvar, ClustOfVar package (version 1.1).


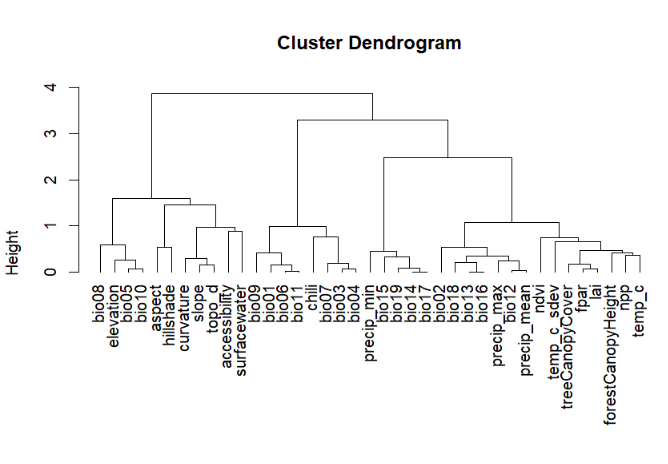


Table S2. Remote-sensing datasets utilized for the selection of model predictors. Remote-sensing data was obtained from Google Earth Engine.

| **Predictor** | **Description** | **Source** | **URL** | **References** |
| --- | --- | --- | --- | --- |
| -Bio 04  - Bio 18 | - Temperature seasonality (Standard deviation * 100)  - Precipitation of warmest quarter | WorldClim BIO Variables V1. Bioclimatic variables derived from the monthly temperature and rainfall. | https://developers.google.com/earth-engine/datasets/catalog/WORLDCLIM_V1_BIO#citations | Hijmans, R.J., S.E. Cameron, J.L. Parra, P.G. Jones and A. Jarvis, 2005. Very High Resolution Interpolated Climate Surfaces for Global Land Areas. International Journal of Climatology 25: 1965-1978. doi:10.1002/joc.1276 |
| -TreeCanopyCover | Time-series analysis of Landsat images in characterizing global forest extent and change | Hansen Global Forest Change v1.8 (2000-2020) | https://developers.google.com/earth-engine/datasets/catalog/UMD_hansen_global_forest_change_2020_v1_8 | Hansen, M. C., P. V. Potapov, R. Moore, M. Hancher, S. A. Turubanova, A. Tyukavina, D. Thau, S. V. Stehman, S. J. Goetz, T. R. Loveland, A. Kommareddy, A. Egorov, L. Chini, C. O. Justice, and J. R. G. Townshend. 2013. "High-Resolution Global Maps of 21st-Century Forest Cover Change." Science 342 (15 November): 850-53. Data available on-line at: https://glad.earthengine.app/view/global-forest-change |
| Precip_mean | Mean precipitation | CHIRPS Pentad: Climate Hazards Group InfraRed Precipitation with Station Data v2.0 | https://developers.google.com/earth-engine/datasets/catalog/UCSB-CHG_CHIRPS_PENTAD | Funk, Chris, Pete Peterson, Martin Landsfeld, Diego Pedreros, James Verdin, Shraddhanand Shukla, Gregory Husak, James Rowland, Laura Harrison, Andrew Hoell & Joel Michaelsen. "The climate hazards infrared precipitation with stations-a new environmental record for monitoring extremes". Scientific Data 2, 150066. doi:10.1038/sdata.2015.66 2015. |
| topo_d | SRTM-derived topographic diversity | Global SRTM Topographic Diversity | https://developers.google.com/earth-engine/datasets/catalog/CSP_ERGo_1_0_Global_SRTM_topoDiversity#citations | Theobald, D. M., Harrison-Atlas, D., Monahan, W. B., & Albano, C. M. (2015). Ecologically-relevant maps of landforms and physiographic diversity for climate adaptation planning. PloS one, 10(12), e0143619 |
| temp_c_sdev | Mean land surface temperature standard deviation | AG100: ASTER Global Emissivity Dataset 100-meter V003 | https://developers.google.com/earth-engine/datasets/catalog/NASA_ASTER_GED_AG100_003#citations | Hulley, G. C., & Hook, S. J. (2008). A new methodology for cloud detection and classification with ASTER data. Geophysical Research Letters, 35(16). https://doi.org/10.1029/2008GL034644 |
| Hulley, G. C., & Hook, S. J. (2009). The North American ASTER Land Surface Emissivity Database (NAALSED) Version 2.0. Remote Sensing of Environment, 113(9), 1967–1975. https://doi.org/10.1016/j.rse.2009.05.005  Hulley, G. C., & Hook, S. J. (2011). Generating Consistent Land Surface Temperature and Emissivity Products Between ASTER and MODIS Data for Earth Science Research. IEEE Transactions on Geoscience and Remote Sensing, 49(4), 1304–1315. https://doi.org/10.1109/TGRS.2010.2063034  Hulley, G. C., Hook, S. J., Abbott, E., Malakar, N., Islam, T., & Abrams, M. (2015). The ASTER Global Emissivity Dataset (ASTER GED): Mapping Earth’s emissivity at 100 meter spatial scale. Geophysical Research Letters, 42(19), 7966–7976. https://doi.org/10.1002/2015GL065564  Hulley, G. C., Hook, S. J., & Baldridge, A. M. (2009). Validation of the North American ASTER Land Surface Emissivity Database (NAALSED) version 2.0 using pseudo-invariant sand dune sites. Remote Sensing of Environment, 113(10), 2224–2233. https://doi.org/10.1016/j.rse.2009.06.005  Hulley, G. C., Hughes, C. G., & Hook, S. J. (2012). Quantifying uncertainties in land surface temperature and emissivity retrievals from ASTER and MODIS thermal infrared data. Journal of Geophysical Research: Atmospheres, 117(D23). https://doi.org/10.1029/2012JD018506  NASA JPL. (2014). ASTER Global Emissivity Dataset, 100-meter, HDF5 [Data set]. NASA EOSDIS Land Processes DAAC. https://doi.org/10.5067/COMMUNITY/ASTER_GED/AG100.003 | | | | |

Fig S2. Example of the sampling layout for the National Forest and Soils Inventory (INFyS) of Mexico implemented for the following forest types: coniferous, broadleaf, coniferous-broadleaf, tropical forest, tropical dry forests, cloud mountain forest and mangroves.


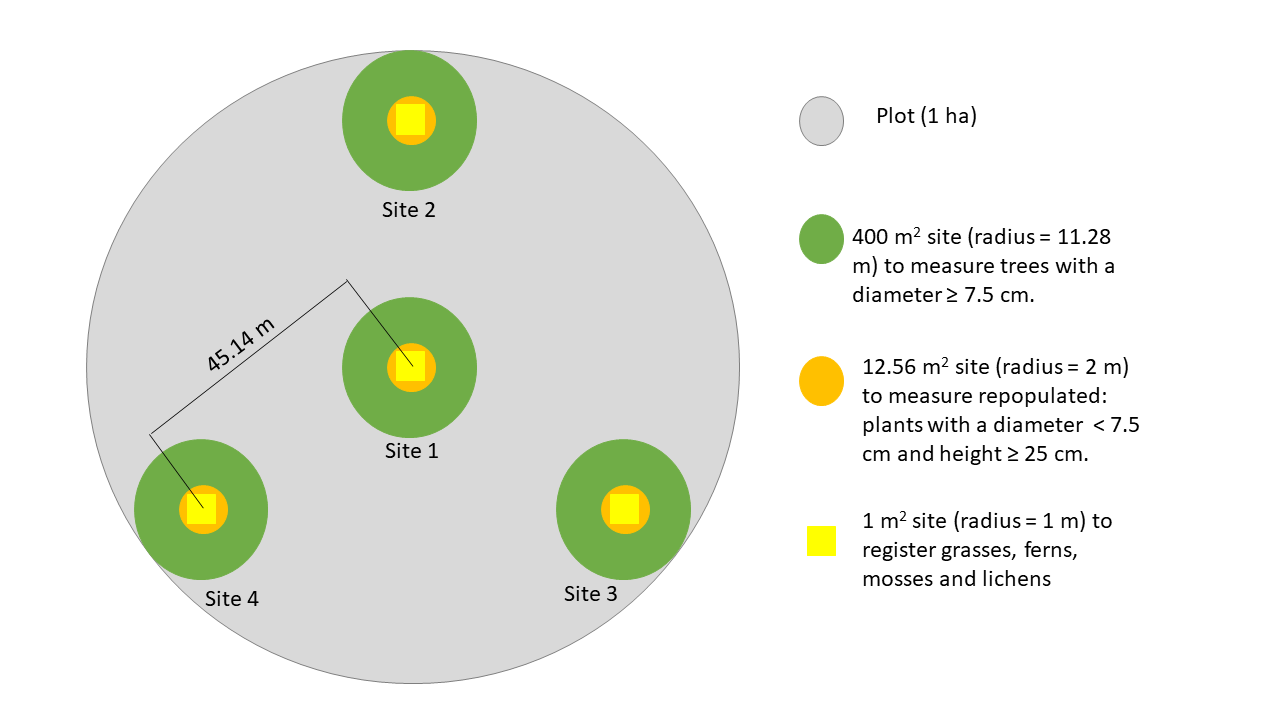


Fig S3. Flow diagram of the methodology to predict the spatial variability of tree height and tree density across Mexico. The green boxes indicate the data source used for model supplies, tree height and density data were used to train and validate the models, respectively. Data sources were harmonized with the covariates at 1000m. Blue boxes indicate the main results of this methodology. CONAFOR: Comisión Nacional Forestal, INEGI: Instituto Nacional de Estadística y Geografía.


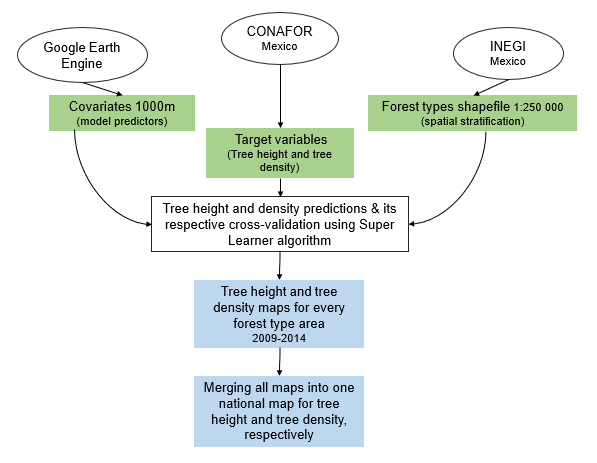


Fig S4. Density plots of a) sampled tree height and b) sampled tree density for each forest type. Data obtained from the National Forest and Soils Inventory (INFyS, CONAFOR 2017).


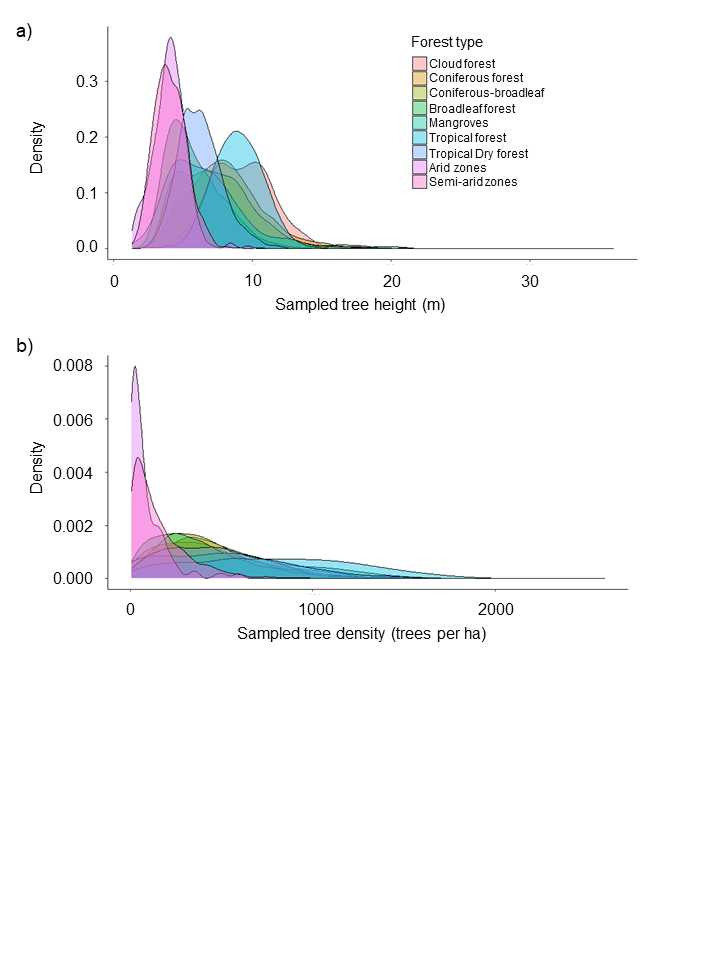


Fig S5. Conditional quantiles plots for the observed vs predicted values of tree height for each forest type.


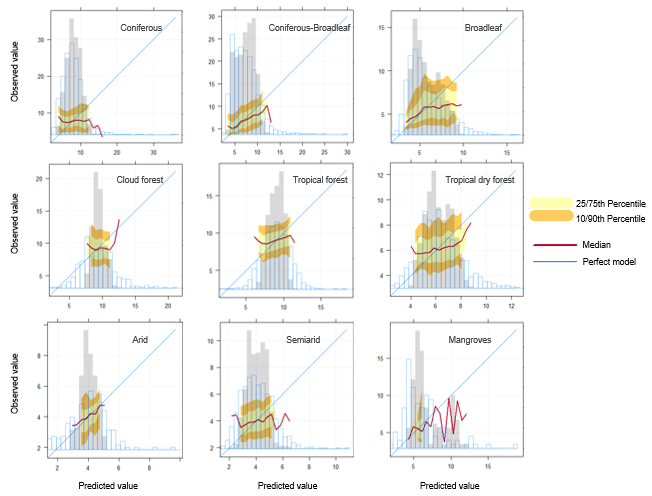


Fig S6. Conditional quantiles plots for the observed vs predicted values of tree density for each forest type.


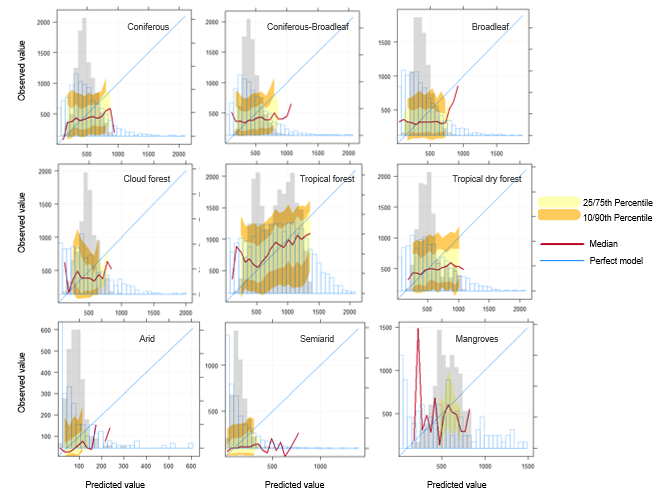

Supplement: Supplementary file 1 — Appendix S1 [file ECE3-13-e10090-s001.docx]
